# Quality of Maternal and Neonatal Care in Albania, Turkmenistan and Kazakhstan: A Systematic, Standard-Based, Participatory Assessment

**DOI:** 10.1371/journal.pone.0028763

**Published:** 2011-12-22

**Authors:** Giorgio Tamburlini, Gelmius Siupsinskas, Alberta Bacci, for the Maternal and Neonatal Care Quality Assessment Working Group

**Affiliations:** 1 Institute for Maternal and Child Health IRCCS Burlo Garofolo and European School for Maternal Newborn Child and Adolescent Health, Trieste, Italy; 2 International Consultant, Basel, Switzerland; 3 Regional Coordinator, Making Pregnancy Safer, World Health Organization Regional Office for Europe, Copenhagen, Denmark; University of Florida, United States of America

## Abstract

**Background:**

Progress in maternal and neonatal mortality has been slow in many countries despite increasing access to institutional births, suggesting deficiencies in the quality of care. We carried out a systematic assessment of the quality of maternal and newborn care in three CEE/CIS countries, using an innovative approach to identify priority issues and promote action.

**Methods:**

A standard-based tool, covering over 400 items grouped in 13 main areas ranging from support services to case management, was used to assess a sample of ten maternity hospitals in Albania, Kazakhstan and Turkmenistan. Sources of information were visit to services, medical records, observation of cases, and interviews with staff and mothers. A score (range 0 to 3) was attributed to each item and area of care. The assessment was carried out by a multidisciplinary team of international and national professionals. Local managers and staff provided the necessary information and were involved in discussing the findings and the priority actions.

**Results:**

Quality of care was found to be substandard in all 13 areas. The lowest scores (between one and two) were obtained by: management of normal labour, delivery, obstetric complications and sick babies; infection prevention; use of guidelines and audits; monitoring and follow-up. Neonatal care as a whole scored better than obstetric care. Interviewed mothers identified lack of information, insufficient support during labour and lack of companionship as main issues. Actions to improve quality of care were identified at facility as well as at central level and framed according to main health system functions.

**Conclusions:**

Quality of care is a key issue to improve maternal and neonatal outcomes, particularly in countries such as CEE/CIS where access to institutional births is nearly universal. Approaches that involve health professionals and managers in comprehensive, action-oriented assessments of quality of care are promising and should be further supported.

## Introduction

Quality of care has recently been recognized as a neglected issue in the international health agenda, particularly with respect to care around childbirth [1]. The existence of a quality gap is the most likely explanation for slow progress towards MDG 4 and 5 where access to institutional births is high, such as in countries of Central and Eastern Europe (CEE) and the Commonwealth of Independent States (CIS). Maternal Mortality Ratio (MMR) decreased in CEE/CIS from 44 deaths per 100 000 live births in 1990 to 21 deaths per 100 000 live births in 2008, corresponding to a decline of 52% in 18 years, and only 5 countries (Estonia, Latvia, Poland, Romania and Turkey) achieved the annual decline of 5.5% needed to reach the MDG 5A target, i. e. a reduction of MMR by 75% [2]. Decrease in the neonatal mortality has been even slower: the average yearly reduction over the last two decades in the Neonatal Mortality Rate (NMR) has been 2% in Central Asia, 3% in Eastern Europe and 3.5% in Central Europe [3]–[5].

Although the regressive social impact of the transition after the breakup of the USSR and the troublesome process of health reform resulted in increasing difficulties in access to care for segments of at risk population [6], [7], the overall picture in CEE/CIS points to the existence of gaps in the quality of care to pregnancy, childbirth and postnatal period [8].

Moving from this assumption, the WHO Regional Office for Europe developed a standard-based assessment tool for maternal and newborn care and supported systematic assessments of the quality of care in a number of CEE/CIS countries, to identify the most critical issues and the priority actions to improve quality. We report the results of systematic assessments carried out in a CEE country (Albania) and two CIS countries (Kazakhstan and Turkmenistan).

## Methods

### Institutional context

The assessment of the quality of maternal and neonatal care is a component of the implementation plan of the WHO Making Pregnancy Safer strategic framework in the European region [9]. which includes technical support to countries to develop or revise policies, laws, norms, regulations and clinical guidelines, to strengthen pre- and in-service training, to make an appropriate use of technologies, to establish a referral system, and to introduce maternal and perinatal audits [10]–[12].

As per WHO mandate, activities were always initiated upon request by Ministries of Health (MoHs), through Biannual Collaborative Agreements (BCAs), and carried out in collaboration between MoHs and a variety of partners. Implementation and scaling up has been ensured through partnership among MoHs, WHO, other UN Agencies (UNICEF, UNFPA), the European Commission, the Asian Development Bank, bilateral aid agencies (USAID, GJZ, Swiss Cooperation, Spanish Agency for Development, and public-private partnerships (Regione Veneto-CariVerona).

### Hospital sample

Based on existing BCAs, three countries were involved in the first round of assessments: Albania, Kazakhstan and Turkmenistan. Three hospitals were initially assessed in Albania: a tertiary referral maternity hospital with neonatal ICU, and two provincial hospitals. The assessment was carried out in February 2009. Four hospitals were assessed in Kazakhstan: a tertiary national referral maternity hospital and three regional maternities, all with NICUs. The assessment took place in November 2009. Three hospitals were assessed in Turkmenistan: a tertiary national referral maternity hospital, a regional and a provincial level maternity, only the first with NICU. The assessment took place in September, 2009. The choice of hospitals to be assessed was made by the MoH, the only requirements for selection being the inclusion of three diverse regions/provinces and at least one referral/teaching hospital.

### Assessment tool

The tool [13] is aimed at: a) guiding the assessors in the collection of information in all key areas which have a major impact on maternal and neonatal outcomes; b) identifying the areas where where poor or substandard care is provided; and c) involving managers and staff at facility level, and MoH in identifying and prioritizing actions to improve quality of care (QoC). The tool is a structured standard-based checklist covering 12 main areas: General Hospital Infrastructure and Services, Maternity ward/nursery and neonatal ward, Care for normal labor and delivery, Routine neonatal care, Caesarean section, Case management of maternal complications, Sick newborn care, Emergency care, Infection prevention and supportive care, Monitoring and follow up, Guidelines audits and team work, and Access to hospital and referral system. Each area includes from 10 to over 50 items for an overall total of around 600 items, a majority of which are devoted to case management. A 13th area covers the quality of information provided to mothers and mother and baby friendly care, relying also on structured interviews with mothers.

The tool utilizes four sources of information: hospital statistics, medical records, direct observation of cases, and semi-structured interviews with staff and with mothers. Interviews with staff are mainly aimed at exploring knowledge and use of guidelines, organizational issues and team work. Interviews with mothers explore patient's satisfaction, obstacles to access and information.

By combining the information from the various sources, a score is attributed to each item and an overall average score to each main area of care. Scores from 3 to 0 are attributed to each item based on the following criteria: 3 = care corresponding to international standards (no need for improvement or need for marginal improvement); 2 = substandard care but no serious hazard to health or violation of human rights (need for improvement); 1 = inadequate care with consequent serious health hazards or violation of women's rights to information, privacy or confidentiality and/or to children's rights, (need for substantial improvement); 0 = very poor care with consequent systematic and severe hazards to the health of mothers and/or newborns, e.g. systematic omission of potentially life-saving interventions or lack of essential safety requisites for key procedures such as caesarean section, blood transfusion, neonatal resuscitation, etc. (need for thorough revision of structure, organization, procedures and case management related to specific items or to the whole area).

The tool has been developed building on the experience gathered with the paediatric hospital assessment tool developed by WHO and extensively used in several countries [14]–[16] and on previous experience on criterion base audits of obstetric care [17].

The reference standards for the case management items are the WHO Integrated Management of Pregnancy and Childbirth (IMPAC) manuals of the global Making Pregnancy Safer programme [18]–[20] and the Effective Perinatal Care (EPC) training package developed by the WHO Regional office for Europe and JSI/USAID [21].

The assessments in Albania, Kazakhstan and Turkmenistan were the first to use the maternal and newborn care quality assessment tool.

### Assessment team

The international assessment team included three experienced professionals covering all key disciplinary backgrounds including obstetrics, midwifery and neonatology, and with international experience as trainers of WHO IMPAC and EPC manuals, plus a team leader. To ensure consistency of methods and scoring, the international teams were the same throughout the assessments carried out in each country, and two out of four members, including the team leader, ensured their participation in at least two countries. The international team was joined by a multidisciplinary national team, selected by the Ministries of Health based on criteria of multidisciplinary composition and professional experience. The number of the national assessors was larger in order to build national capacity in conducting the assessment.

### Assessment methods

The tool was sent in advance to countries through WHO Country offices, in order to provide translation (Russian and Albanian). An information was sent by the Ministries of Health to the managers of the hospitals selected for the assessment, with the request to fill in the sections of the tool covering general information on patient flow, structure, staff, availability of drugs supplies and equipment A one-day workshop was organized prior to the visits to ensure that all members of the national team were familiar with contents and methods.

The visit started with an initial briefing to key staff and managers on the objectives and methods of the assessment, and included all relevant services, from pharmacy to laboratory, and units, from admission to intensive care. The duration varied from 6 to 8 hours depending on the size of the hospital. The assessors met after the visit to discuss findings, attribute scores and prepare the feedback meeting, which was held at the end of the assessment, usually the day after, with the participation of hospital managers, heads of units and support services, to present and discuss findings and suggested actions. Actions were divided into actions to be taken at local and central level and were framed according to the main health system functions (governance and stewardship, financing, human resources and infrastructure, service delivery). The findings were then presented to MoH and key partners. A follow-up assessment has been planned in all countries.

### Analysis of findings

Given the essentially qualitative nature of the exercise, no statistical analysis was performed beside the calculation of average scores for each main area of care. A detailed analysis of the findings was done in each institution and the findings were then aggregated for this paper.

### Ethics approval

The paper reports an audit of the quality of maternal and neonatal care in Albania, Turkmenistan and Kazakhstan. The activity was planned together with and was approved by the Ministry of Health in each country within the Biennial Country Agreements with the WHO Regional Office for Europe. There are no Institutional Review Boards in the hospitals included. However, the hospital directions received and approved the assessment tools and methods prior to the visit. Mothers were anonymously and confidentially interviewed. Written consent was not asked because most women would not accept to be involved in an interview if they had to sign papers. External assessors and local staff agreed on the fact that the best way to obtain free opinions from women was through an informal approach by people that qualified themselves as health professionals external to the institutions, clarified the purpose of the interview and of the whole audit activity, guaranteed the anonymity, and then asked for verbal consent.

## Results

The main characteristics of the countries and hospitals involved in the assessment are described in table 1. The assessment covered facilities providing care to an average of 10 to 15% of the total number of deliveries per year (2009) in each of the three countries. The number of deliveries per hospital ranged from 1200 to over 5000.

**Table 1:** Characteristics of the countries and of the maternity hospitals involved in the assessment.

	Albania	Kazakhstan	Turkmenistan
Population (2009)	3.172.000	15.522.000	4.899.000
GNP (USD PPP, 2008)	6.000	8.800	3990
MMR, last available estimate and change 1990–2008 [1]	31 per 100.000 (−35%)	45 per 100.000 (−42%)	77 per 100.000 (−16%)
NMR, last available estimate and change 1990–2010 [3]	3.7 per 1000 (−59%)	15.3 per 1000 (31%)	13.7 per 1000§ (−59%)
Institutional deliveries as % of total deliveries	>97%	>97%	>97%
Number of hospitals assessed	3 (1 Referral)	4 (1 Referral)	3 (1 Referral)
Number of births in the hospitals assessed (% of total births in country)	8.540 (16%)	32. 810 (10%)	9.990 (10%)

§ Confidence intervals for Turkmenistan are extremely wide (8.0–21.9 per 1000)^3^.

Overall, the quality of care was found substandard in all areas. The average score was particularly low (average score between one and two) in 7 out of 13 areas (table 2), including care of normal labour and delivery, care of obstetric complications, care of sick newborn babies, infection prevention, use of guidelines and auditing practices, monitoring and follow-up, mother and baby centred care. There were no areas with an overall average score equal or under one, although a few items within each area scored less than 1. Neonatal care, including essential care and care of sick newborns (2.0 and 1.9 respectively) scored as a whole better than care for normal labour and delivery and maternal complications (1.5 and 1.5) and care in one intensive care unit scored three. Overall, the average scores were slightly higher in referral hospitals with respect to other hospitals (2.2 versus 1.9), otherwise showed relatively little variation across the sample of ten hospitals. Seven hospitals had at least one area of care classified as standard, two had three and one four, the latter all being referral hospitals.

**Table 2:** No. of maternity hospitals showing standard care and average scores in the areas covered by the assessment.

Areas	No. of maternity hospitals showing standard care (out of a total of 10) in each main area	Average score (all 10 hospitals)
Infrastructure, equipment and supplies	1	2.0
Maternity and neonatal ward	2	2.1
Care for normal labour and delivery	2	1.5
Routine neonatal care	4	2.0
Caesarean section	2	2.0
Maternal complications	0	1.5
Sick newborn care	1	1.9§
Emergency preparedness	2	2.2
Infection prevention and supportive care	0	1.0
Monitoring and follow-up	0	1.4
Guidelines, auditing and team work	0	1.3
Access to hospital	0	2.0
Mother and baby-centered care	0	1.1

§ Two maternity hospitals did not have NICUs, so only non-intensive care was assessed.

**Figure 1:**
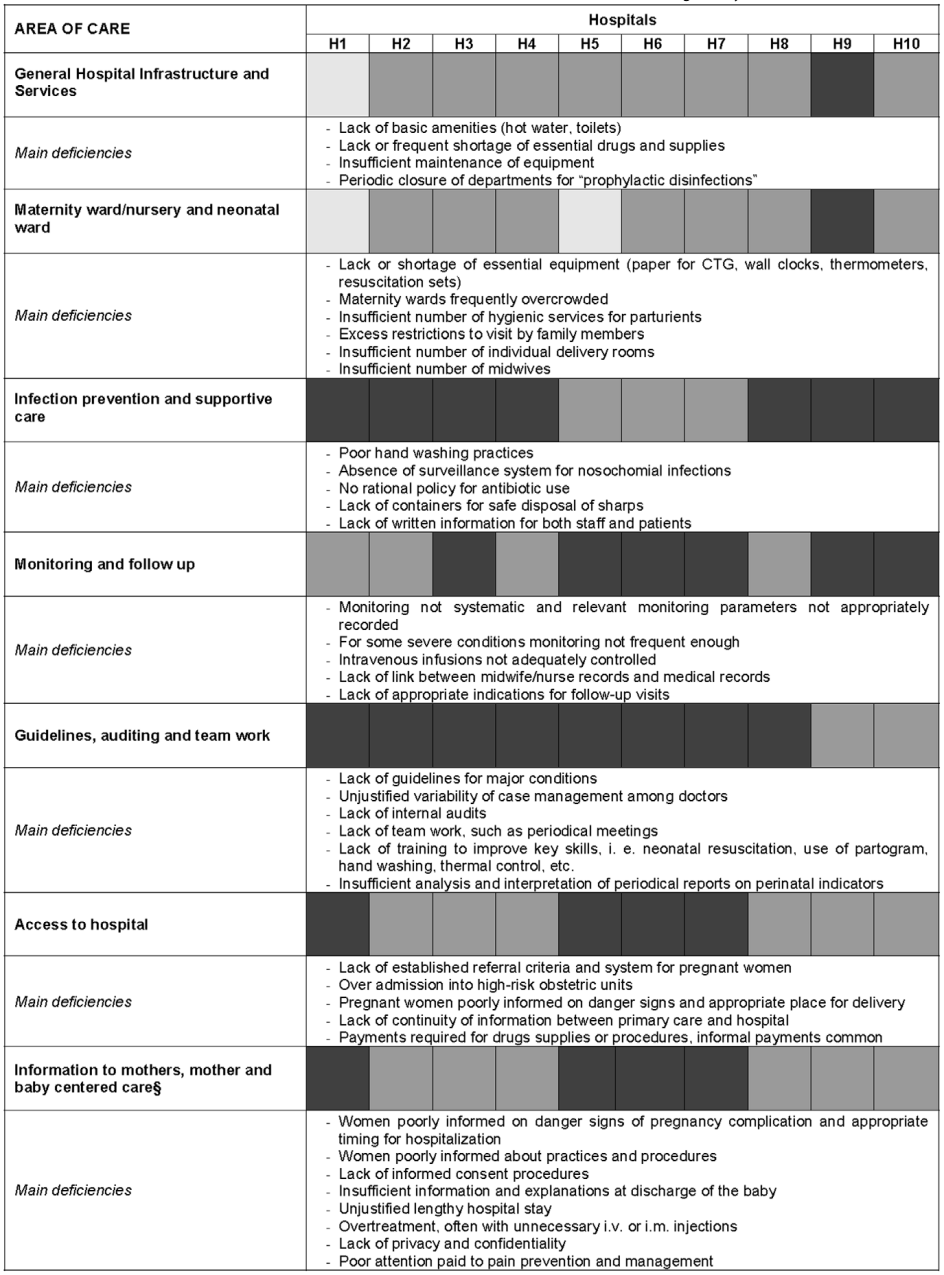
Quality of maternal and neonatal care. Main deficiencies in infrastructural and procedural issues.

**Figure 2:**
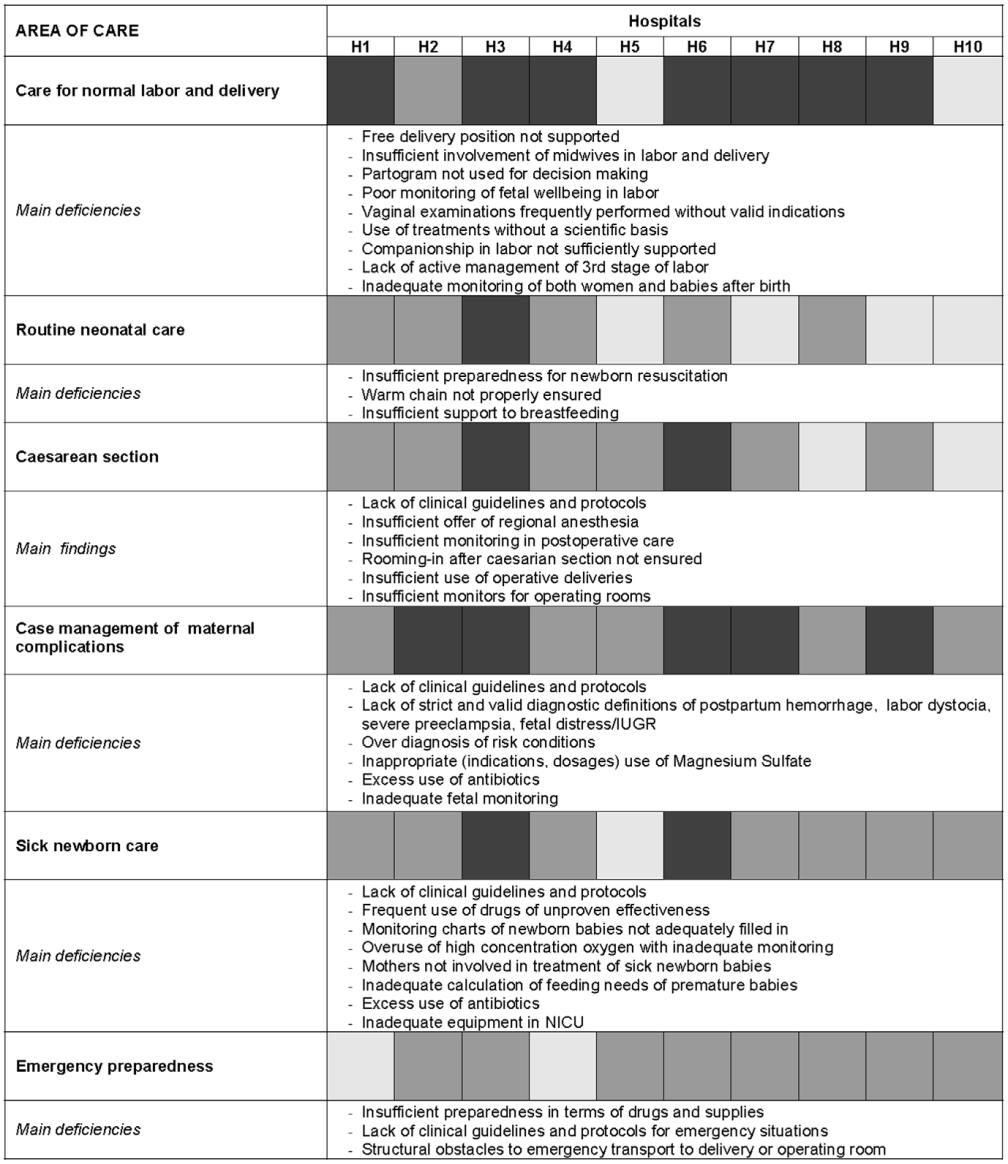
Quality of maternal and neonatal care. Main deficiencies in case management. ▪ = 3: care corresponding to international standards (i.e. no need for improvement or need of marginal improvements); ▪ = 2: substandard care but no significant direct hazard to health or violation of human rights (need for improvement to reach standard care); ▪ = 1: inadequate care with consequent serious health hazards or violation of women's to information, privacy or confidentiality and/or to children's rights, e.g. omission of evidence based interventions or information with consequent risk for physical integrity (need of substantial improvement to reach standard care).

The average scores for each area in all ten maternity hospitals and the main deficiencies identified in each area of care are shown in figure 1 and 2, the former illustrating infrastructural and procedural issues and the latter case management issues. Unjustified lengthy admissions, over diagnosis of risk conditions, inappropriate use of drugs, often with unnecessary intravenous or intramuscular treatments for both mothers and babies, were widespread. Inadequacies in care of normal labor, such as poor foetal monitoring, and poor management of second and third stage of labour, coexisted with deficiencies in the case management of the most serious and threatening conditions, such as postpartum hemorrhage, labor dystocia, severe pre-eclampsia, and prematurity, especially regarding nutritional needs and oxygen administration. Inadequate attention paid to privacy and confidentiality and poor attention paid to pain prevention and management were also common. Referral criteria and systems and communication between levels of care were also lacking.

Interviews to an average of 12 to 20 women in each country (pregnant women or mothers of admitted children) provided insights into a number of issues including access to hospital, direct and indirect costs incurred, perceived quality of care and information received at admission, during hospital stay and at discharge. The lack of adequate information was the most common complaint, mentioned by almost all the interviewed mothers, followed by insufficient support during labour and for initiation of breastfeeding and by lack of companionship during labour.

Interviews and discussion with staff confirmed the findings of the assessment, and emphasised a number of problems faced by the staff, such as low salaries, absence of incentives, conflict among existing guidelines, lack of training, poor laboratory support and periodic problems in the availability of some drugs and supplies. The participatory peer to peer approach was very well received by health professionals, who acknowledged the novelty of the method with respect to the mainly inspectional and non participatory assessments experienced previously.

There was evidence, from interviews with staff and women, of out-of-pocket disbursements, either to buy drugs which were not available at the hospital pharmacy, or for informal payments to staff. The latter circumstance was obviously under reported, as the interviews contents and setting were not specifically designed to investigate this aspect.

The assessment process led to the identification of priority actions at facility level as well as central, ministerial level. Some of the actions identified at facility level were very specific, focusing for example on improving the availability of drugs, supplies and amenities or revising some organizational procedures. However, in view of the findings which largely overlapped, the suggested actions largely overlapped. They are summarized in table 3, according to four main health system components: stewardship and governance, resource generation, financing and service delivery [22]–[23].

**Table 3:** Main proposed actions to improve the quality of care at facility and central level, by health system function [23].

Health system function	Facility level	Central level
**Stewardship and governance**	1) Develop and implement a detailed action plan, including responsibilities and time line, to address the main quality gaps that are suitable of action at facility/unit level; 2) Run periodic hospital and department team meetings to update protocols, analyze and discuss patient flow and perinatal indicators; 3) Promote case reviews and perinatal audits	1) Define a process, if necessary with international technical support, for the development/revision of clinical guidelines based on international standards; 2) Remove/modify norms and regulations that are in contrast with international guidelines; 3) Ensure access to internet-based knowledge management tools in languages accessible to health professionals; 4) Set up a mechanism of periodical (yearly) data review involving district and hospital managers
**Financing**	1) Introduce cost indicators (drug use, lab use, indications for admission and treatment) to identify areas for potential savings; 2) Enforce regulations regarding private professional work and informal payments	1) Improve budget decentralization and accountability; 2) Consider performance-based financial incentives to facilities, departments and health professionals
**Resource generation and management**	1) Plan internal and external training opportunities for the medical and midwifery/nursing staff based on the priority areas identified by the assessment; 2) Promote access to internet for all staff; 3) Enhance the role of nurses and midwives in monitoring, case management and information	1) Revise training curricula for health professionals to introduce key concepts of evidence based medicine, patient communication, quality improvement and clinical audit; 2) Improve procurement and distribution availability of essential medical technologies; 3) Improve the availability of essential laboratory investigations
**Service Delivery**	1) Make agreements with peripheral hospitals for referral of at risk cases; 2) Ensure adequate record keeping and monitoring, improve communication with patients and families assigning specific roles to medical staff, nurses and midwives; 3) Improve privacy and support patient choices during labour and delivery and post-partum care; 4) Promote a friendly and caring environment for both mothers and babies	1) Establish criteria for a perinatal referral system, including structural, equipment and staffing requisites for each level of care, criteria for *in utero* transfer, and equipment and skills for neonatal transport; 2) Develop guidelines and tools to ensure continuity of care, including information systems mothers and children's individual records; 3) Develop together with professional societies written information for mothers on common conditions regarding pregnancy, delivery and neonatal care

## Discussion

The assessment carried out in a sample of ten maternity hospitals in Albania, Kazakhstan and Turkmenistan showed that the quality of care for mothers and newborn babies was substandard in all the areas explored. The poorest performances were observed in key aspects such as the management of normal labour and delivery, maternal complications and sick newborn babies, infection control, availability and use of appropriate guidelines, monitoring and follow-up, audit systems and patient-centred care. The findings were remarkably similar across the sample, which is not surprising given the similarities in both health systems and mainstream ideology in the three countries prior to transition [7], [8], [10]–[12].

Specific deficiencies potentially leading to serious health hazards for both mothers and babies were found. Disregard of women's right to information, privacy or confidentiality was also common, as a consequence of lack of recognition of such rights among managers and health professionals and low awareness of their implications for health and wellbeing of both mothers and babies. Over-diagnosis of risk conditions and complications leading to over-admission, polypharmacy and consequent hazards was also common, confirming previous reports from CIS countries and regarding paediatric care [14]. Implications of unnecessary admissions and treatments include unjustified costs for both households and health systems, also taking into account the evidence of out-of-pocket disbursements by patients. Infrastructure, staffing, equipment, availability of essential drugs and supplies were often found substandard and sometimes frankly inadequate. However, as previously reported in CEE/CIS countries and elsewhere [14]–[15], [24], these deficiencies did not seem to represent the main limiting factor to ensure safety, effectiveness, and patient responsiveness, i.e. the main dimensions of quality care [25].Widespread and important weaknesses were found in cross-cutting components of care which do not require sophisticated infrastructure or equipment, such as the existence and utilization of updated guidelines and protocols, case reviews and audits.

Neonatal care scores were slightly higher than obstetric care scores. This is likely to be due to the different amount of inputs received. International agencies have focussed earlier on areas such as breastfeeding and neonatal resuscitation, although the high turnover of health professionals may have reduced the impact of inputs. However, there were cases of facilities where breastfeeding promotion was satisfactory but other key aspects of neonatal care were neglected. Both facts emphasize the need for comprehensive training in perinatal care and for a broader scope of the baby friendly hospital concept. There was also an example of optimal care in one NICU, reflecting both excellent local leadership and a long record of international collaboration.

While many of the observed deficiencies can be effectively addressed at local level, others, such as those regarding referral system, standards and norms, clinical guidelines, equipment, training, require primarily action at MoH and/or at government level.

Among the actions that can be implemented at local level there are the development of local protocols, the respect of women's privacy confidentiality and choices, the meaningful use of existing data and information including periodic case review and audits, improved infection control, better clinical monitoring and improved information to women during hospital stay and at discharge. Among the actions that need action at national level there are the establishment of a referral system, including standards of staffing equipment and laboratory support, criteria for *in utero* transfer; improved pre-service training including principles of evidence based medicine, quality improvement and communication skills, a more courageous decentralization of budgetary responsibilities, including the possibility to reinvest locally the savings deriving from rational use of drugs and to introduce performance-based rewarding systems [26].

Our findings further support the view, emphasized by the WHO EURO MPS strategic approach that the efforts to improve quality need to be strengthened in the Region. The implications of improved quality of care in the perinatal period are not limited to the reduction of maternal and neonatal mortality, but include the minimization of long term sequelae for both women and newborn babies, improved nutrition and attachment.

The quality gap is not confined to CEE/CIS countries and its is increasingly recognized as a priority [1], [29]. Many systems for quality assessment and improvement have been proposed, implemented and evaluated, in Europe and elsewhere [27]–[29]. A common feature of many such systems is that they require an important financial investment and a the availability of specifically trained professionals, both requisites being difficult to meet in under-financed health systems. Furthermore, most approaches to quality improvement and related certification and accreditation processes focus on infrastructure, equipment, written procedures and protocols rather than on actual case management. The involvement of local professionals is limited and the feedback not immediate, with the result that these approaches are far more popular among managers than among professionals. Participatory approaches based on peer review, such as the one we described, maternal and perinatal death and near-miss reviews and criterion-based audits [12], [17] seem more promising, particularly if the main purposes is, as it should be, to develop professionalism as a solid basis for continuous quality improvement.

We made an attempt to combine the benefits of a peer-review approach aimed at motivating the professionals involved at facility level with the need for a systematic approach to identify the actions also at system level. Based on our experience, this combination is effective in building awareness on quality issues and promoting change at facility level and at the same time at the national level. Our supportive and participatory approach was particularly welcome by professionals who had previously experienced only bureaucratic controls and sanctions.

Table 4 illustrates the conceptual framework of our approach and lists the main requisites and challenges for implementation.

**Table 4:** Quality of Maternal and Newborn Care assessment tool: key features, requisites and challenges.

Main health System Components	Main features of the tool	Requisites and Challenges
**Standards**	1) WHO IMPAC standards and guidelines; 2) EPC training modules	1) Revised and updated national guidelines and methodological support to the development of local protocols
**Measurements**	1) Comprehensive assessment of all aspects of care including infrastructure, drugs and supplies, service organization, clinical management, medical records, team work, links with other levels of care; 2) Patients' views collected	1) Adequate planning for the assessment: information to health facilities about scope and purpose, adequate time for introduction, assessment, interviews and feedback.
**Strategies**	1) Professionals involved in assessing their practice, identifying problems and solutions and defining an action plan; 2) Peer to peer discussion aimed at identifying critical areas, root causes and feasible solutions; 3) Suggested actions to be taken at local level and at central level	1) Availability of technically competent, authoritative and independent assessment team; 2) Attitude and experience for a sensible, supportive peer-to-peer approach
**Driving forces**	1) Motivation of health professionals and hospital managers; 2) Advice and example provided by competent and authoritative professionals; 3) Possible link with performance-based or accreditation mechanisms	1) Development of accreditation/certification mechanism or performance-based bonuses for facilities and individuals; 2) Development of strong and independent professional associations

We acknowledge the limitations of our approach. First, although a scoring system based on clear criteria was adopted and the continuity of the evaluation team ensured, the comparability of the assessment across health facilities cannot be completely guaranteed. Second, although the sample of hospitals covered a significant proportion of deliveries, it remains a convenience sample based on Ministry of Health indications, with possible bias towards the better performing institutions. However, we emphasize that the main purpose of the assessment was not to guarantee the maximum of accuracy and reproducibility, but rather to prompt quality improvement cycles through a participatory identification of key deficiencies and relevant actions to address them. The assessment was an opportunity for capacity building at national level, through the establishment a national team of assessors who got familiar with the assessment tools and methods, and are now able to lead or contribute to further assessments. It supported the introduction of international guidelines and protocols, providing concrete and immediate examples of the relevance of the guidelines to specific case management issues. Finally, it contributed to introducing the concept of peer review among hospital managers and health professionals, thus reinforcing the messages introduced through the near miss case reviews. By providing comprehensive and systematic assessment on all aspects of care, the tool can be used for accreditation and certification systems, and its items could be used as criteria for certification. Subsequent assessments could monitor progress in specific areas until all the main criteria for certification are met. To provide further evidence of effectiveness and to assess the potential to serve for accreditation purposes, follow-up evaluations are planned within 24 months from the first assessment visits.

### Conclusions

In CEE and CIS countries, where the coverage of antenatal and delivery care is almost universal, accelerated progress in maternal and neonatal outcomes requires better access to antenatal and perinatal care for population groups that are inadequately served or excluded from care but also and improving the quality of care for all [6], [8]–[9]. International agencies and donors have so far focussed their efforts on development and dissemination of evidence based guidelines on delivery care and early neonatal care and on related training, without paying sufficient attention to systematic assessment of quality of care and introduction of quality improvement systems [1]. This situation is not confined to the CEE/CIS Region and the quality gap needs to be more effectively addressed in all countries to achieve MDG 4 and 5. Approaches as the one described in this paper are able to identify priorities through an engagement of health professionals and hospital managers should receive further support. They should be seen as key components of comprehensive strategies to improve demand, access and quality of maternal and newborn care.
